# Evaluation of Gingival Depigmentation Using Different Surgical Techniques and Prevention of Repigmentation With Vitamin C: A Clinical Study

**DOI:** 10.7759/cureus.76925

**Published:** 2025-01-04

**Authors:** Rizwan M Sanadi, Revati S Deshmukh

**Affiliations:** 1 Periodontology and Oral Implantology, Dr. G. D. Pol Foundation's Y.M.T. Dental College and Hospital, Navi Mumbai, IND; 2 Oral Pathology and Microbiology, Bharati Vidyapeeth Dental College and Hospital, Pune, IND

**Keywords:** gingival depigmentation, gingival melanin hyperpigmentation, laser depigmentation, melanin, scalpel depigmentation, vitamin c

## Abstract

Background

Gingival depigmentation is a cosmetic periodontal plastic surgical procedure for removing or reducing unesthetic melanin hyperpigmentation-the reappearance of melanin pigmentation after gingival depigmentation has been reported. Vitamin C has been routinely used for the management of hyperpigmentation in dermatology. However, very few studies have been conducted on the use of Vitamin C for the management of gingival hyperpigmentation. Further, the use of Vitamin C to prevent the recurrence of pigmentation after its surgical removal has not been reported. Hence, the present study was conducted.

Aim

The aim of the study is to evaluate gingival depigmentation by different surgical techniques and the prevention of repigmentation using Vitamin C.

Methods

Sixty-four sites in subjects aged 20-45 years with chief complaints of black-looking gums were selected. Scaling and root planning were done. Gingival depigmentation was performed by scalpel at half the sites (32 sites) and by laser technique at the other half sites (32 sites) under adequate local anesthesia. The gingival pigmentation index (Dummett-Gupta oral pigmentation index (DOPI)), visual analog scale (VAS), and surface area of pigmentation were assessed. Vitamin C was administered two weeks after surgical depigmentation and then at monthly intervals for six months.

Results

There was a statistically significant decrease in the DOPI score, the surface area of pigmentation from baseline to one year (p < 0.000), and the VAS score at laser-treated sites as compared to scalpel-treated sites at one month (p < 0.000).

Conclusion

Laser depigmentation is minimally invasive and preferred. Recurrence of gingival melanin pigmentation can be minimized by the administration of Vitamin C.

## Introduction

A beautiful smile reflects a feeling of joy, success, and self-confidence. In addition to the shape, position, and color of teeth, the color of the gingiva also plays an important role in an esthetic smile [1]. A healthy gingiva may appear pale pink, pink, or deep bluish-purple in color [2]. It is greatly influenced by the presence of melanin pigmentation. Melanin, a non-hemoglobin-derived brown pigment, is responsible for the normal pigmentation of the skin, gingiva, and oral mucous membrane. Melanin pigmentation can occur at any age, irrespective of gender, and contributes to racial pigmentation [3].

Melanin hyperpigmentation of gingiva can be seen as patches of light brown to dark brown or as a band of irregularly shaped brownish discoloration [2]. It is produced by melanocytes, which are present in the innermost layer of the epithelium. Melanin hyperpigmentation of gingiva occurs due to excessive deposition of melanin [4]. Physiological pigmentation is probably genetically determined and partially related to mechanical, chemical, and physical stimulation. Melanin hyperpigmentation can be attributed to both exogenous and endogenous factors. Endogenous pigmentation may be seen in diseases like Addison’s disease and type 1 neurofibromatosis. Exogenous pigmentation may be caused by metals such as copper, mercury, amalgam, graphite, silver, bismuth, arsenic, lead, and gold and some kinds of tattoos.

Gingival depigmentation is a periodontal plastic surgical procedure that aims at removing or reducing this hyperpigmentation by various techniques [5]. Various depigmentation techniques include surgical methods (a. scalpel surgical technique, b. bur abrasion method, c. electrosurgery, d. cryosurgery, e. lasers, and f. radiosurgery), chemical methods, and methods aimed at masking the pigmented gingiva (free gingival graft, acellular dermal matrix allograft) [6]. The gingival phenotype and the degree of pigmentation determine the type of depigmentation technique to be performed. However, the reappearance of the gingival melanin pigmentation after a period of gingival depigmentation has been reported and termed as repigmentation.

Vitamin C is nothing but ascorbic acid, a water-soluble essential nutrient required for the formation of collagen and the conversion of dopamine to norepinephrine [7]. In normal healthy conditions, it provides protection against ultraviolet radiations, strengthens the skin, and helps to modulate immunity. It is a powerful antioxidant and has also been used in the treatment of cancer and the removal of hyperpigmented spots on the skin [8].

In dermatology, Vitamin C has been routinely used as a depigmenting agent [9]. Vitamin C has been used as a non-surgical agent for depigmentation of gingiva. This highlights an opportunity for research in the field of periodontology and oral health. The use of Vitamin C to prevent the recurrence of pigmentation has not been reported as yet. Vitamin C has antiaging and antioxidant properties and has shown a marked benefit in dermatology. Therefore, its chances of showing similar activity in gingival epithelium seem likely. Therefore, this study aimed to evaluate gingival depigmentation by different surgical techniques and the prevention of repigmentation using Vitamin C.

## Materials and methods

An experimental study was designed. The protocol of this study was approved, and permission was obtained from the Research Committee and Ethics Committee of the Institute with Registration number EC/NEW/INST/2021/MH/0029 dated November 25, 2022. The study protocol was registered at the Clinical Trial Registry of India (CTRI) with no. CTRI/2021/07/035019 on July 20, 2021.

Systemically healthy individuals in the age range of 20-45 years with chief complaints of black-looking gums having physiological gingival melanin hyperpigmentation (score ≥ 2 of Dummett-Gupta oral pigmentation index (DOPI) 1964) [10] were included in the study. Individuals in this age range are concerned about their esthetics and self-motivated for treatment. Subjects taking medications that may cause gingival melanin hyperpigmentation, smokers, drug addicts, alcoholics, those with autoimmune or endocrine disorders, and pregnant and lactating mothers were excluded from the study.

A total of 64 sites diagnosed with physiological gingival melanin hyperpigmentation in maxillary and/or mandibular arches were divided into four groups with a sample size of n = 16 sites per group. An equal number of maxillary and mandibular arches were included. A simple random sampling technique with the chit method was used in consultation with the statistician. Sites were grouped as Site A1 (scalpel technique), Site A2 (scalpel technique + Vitamin C), Site B1 (laser technique), and Site B2 (laser technique + Vit C).

The clinical parameters assessed in the study were the following:

1. DOPI 1964 [10] was used to assess the degree of gingival melanin pigmentation. Score 0 indicated no pigmentation; Score 1 mild pigmentation; Score 2 moderate pigmentation; and Score 3 severe pigmentation. It was recorded at baseline and after two weeks, one month, three months, six months, nine months, and one year postoperatively.

2. The visual analog scale (VAS) [11] was used to rate the degree of pain on the 10-cm horizontal scale. The right endpoint of the pain scale was designated as ‘‘no pain,’’ and the left endpoint was marked as ‘‘worst pain imaginable.’’ The rating of 0-3 was recorded as mild pain, 4-7 as moderate pain, and 8-10 as severe pain. The VAS score was recorded one hour after the depigmentation surgery. Subsequently, after two weeks, when the subject reported for follow-up, the VAS scores were recorded at Sites A1 and B1 and after two weeks, one month, three months, and six months at Sites A2 and B2 postoperatively.

3. The surface area of pigmentation was measured using the ImageJ analysis software (National Institutes of Health, Bethesda, MD, US) [12]. The area of pigmentation from the mid-buccal area of the right first premolar region to the mid-buccal region of the left first premolars was traced on the intraoral photographs. To minimize the chances of error in recording, each surface area was traced thrice, and measurements were noted. The average value of the three measurements was considered. The surface area of pigmentation at the sites was assessed at baseline and postoperatively at two weeks, one month, three months, six months, nine months, and one year.

Methodology

A detailed case history of all the selected subjects was recorded. Signed informed consent was obtained after explaining the study in detail. All subjects received thorough scaling and root planning with ultrasonic scalers and hand instruments. Subjects were motivated to maintain good oral hygiene. Subjects were instructed to refrain from colored foods and mouthwashes. Photographs were taken for all subjects in the same position. Gingival depigmentation surgery was performed one week after scaling and root planing. The study was carried out in two phases: Phase 1: Surgical Phase and Phase 2: Administration of Vitamin C.

Phase 1: Surgical Phase

**Figure 1:**
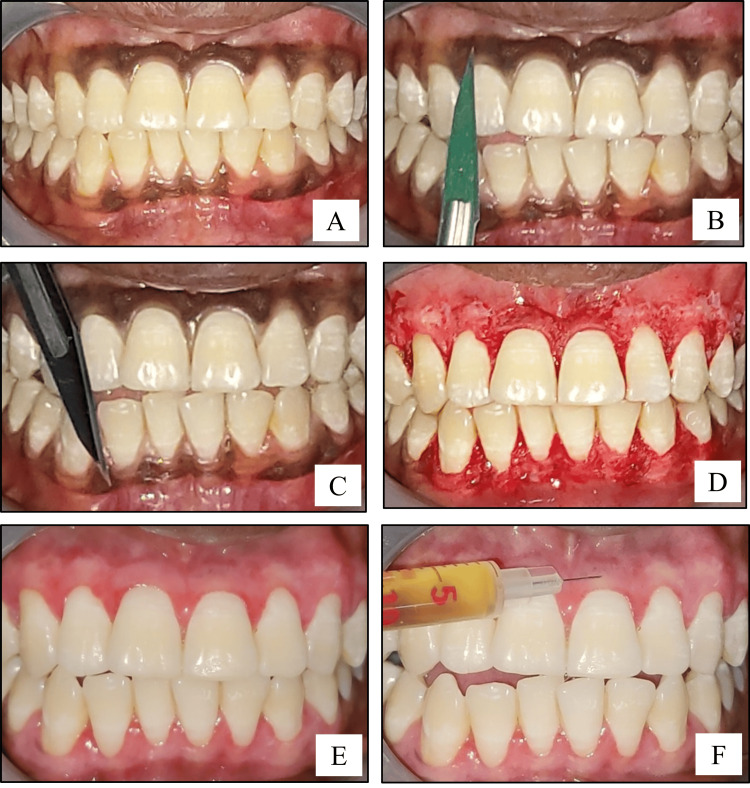
Surgical procedure (scalpel technique): (A) intraoral baseline photograph showing generalized melanin pigmentation; (B,C) intraoral photograph showing the intraoperative view of depigmentation being performed by the scalpel technique on maxillary and mandibular gingiva, respectively; (D) intraoral photograph showing immediate postoperative view after depigmentation; (E) intraoral photograph showing two weeks postoperative view after depigmentation; (F) intraoral photograph showing the administration of Vitamin C into the gingiva with the help of an insulin syringe.

Under aseptic precautions and after adequate local anesthesia (2% lignocaine hydrochloride solution with adrenaline 1:80,000 dilution), surgical depigmentation of the gingiva was performed using surgical scalpel blade no. 11 along with Bard-Parker handle no. 3. Intraoperative bleeding was arrested by pressure application with the help of sterile saline-soaked gauze. The pigmented epithelial layer, along with the thin underlying connective, was excised. Magnification glasses were used to ensure complete removal of the pigmented epithelium (Figures 1A-1E). Non-eugenol periodontal dressing (Coe-Pak (GC International AG, Luzern, Switzerland)) was placed for a duration of one week.

**Figure 2:**
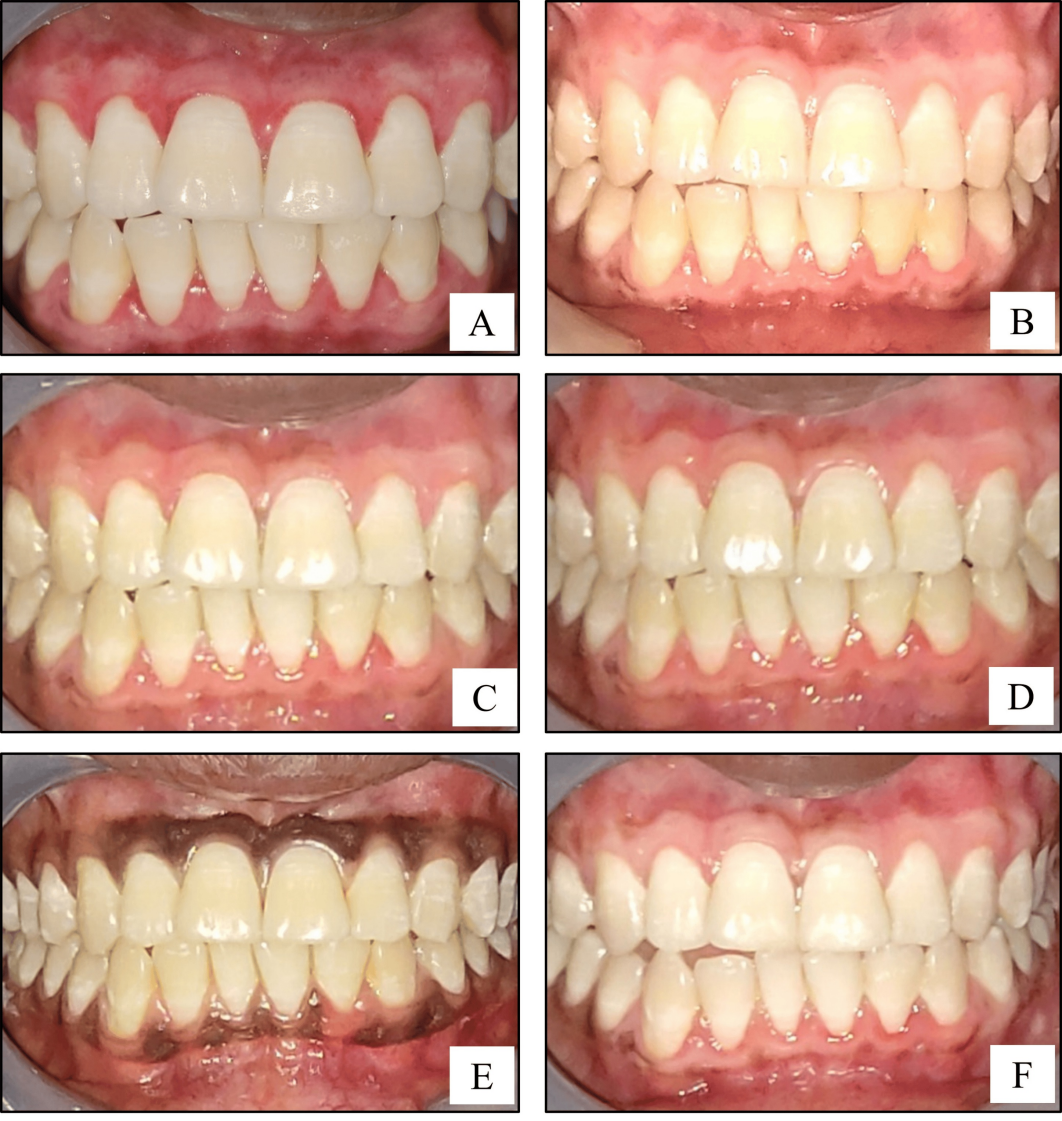
Surgical procedure (scalpel technique-follow-up): (A) intraoral photograph showing one month postoperative view of the depigmented gingiva; (B) intraoral photograph showing three months postoperative view of the depigmented gingiva; (C) intraoral photograph showing six months postoperative view of the depigmented gingiva; (D) intraoral photograph showing nine months postoperative view of the depigmented gingiva; (E) intraoral photograph showing baseline preoperative view of the pigmented gingiva; (F) intraoral photograph showing one year postoperative view of the depigmented gingiva.

As for the laser procedure, the diode laser of wavelength 810 nm at 1-1.5 W power delivered through a flexible fiber optic tip of 400 μm diameter was used in a continuous contact wave mode. A laser safety protocol was followed. Safety glasses were worn, and plastic mouth mirrors and probes were used. The pigmented epithelial tissue was ablated in a brushstroke pattern till the entire pigmented layer was removed. A sterile saline-soaked gauze was used to remove the remnants of the ablated tissue. Complete removal of the pigmented epithelium was ensured with the help of magnification glasses (Figures 2A-2E).

All the subjects were given postoperative instructions. They were advised to avoid mechanical plaque control at the surgical site for one week and to resume the same thereafter. They were instructed to use chlorhexidine mouthwash twice daily for two weeks. An analgesic (ibuprofen 400 mg s.o.s.) was prescribed, and subjects were asked to take it if pain arose.

They were recalled after one week for postsurgical evaluation. Subsequently, postoperative follow-up evaluation was done at the predetermined time intervals mentioned above.

Phase 2: Administration of Vitamin C

**Figure 3:**
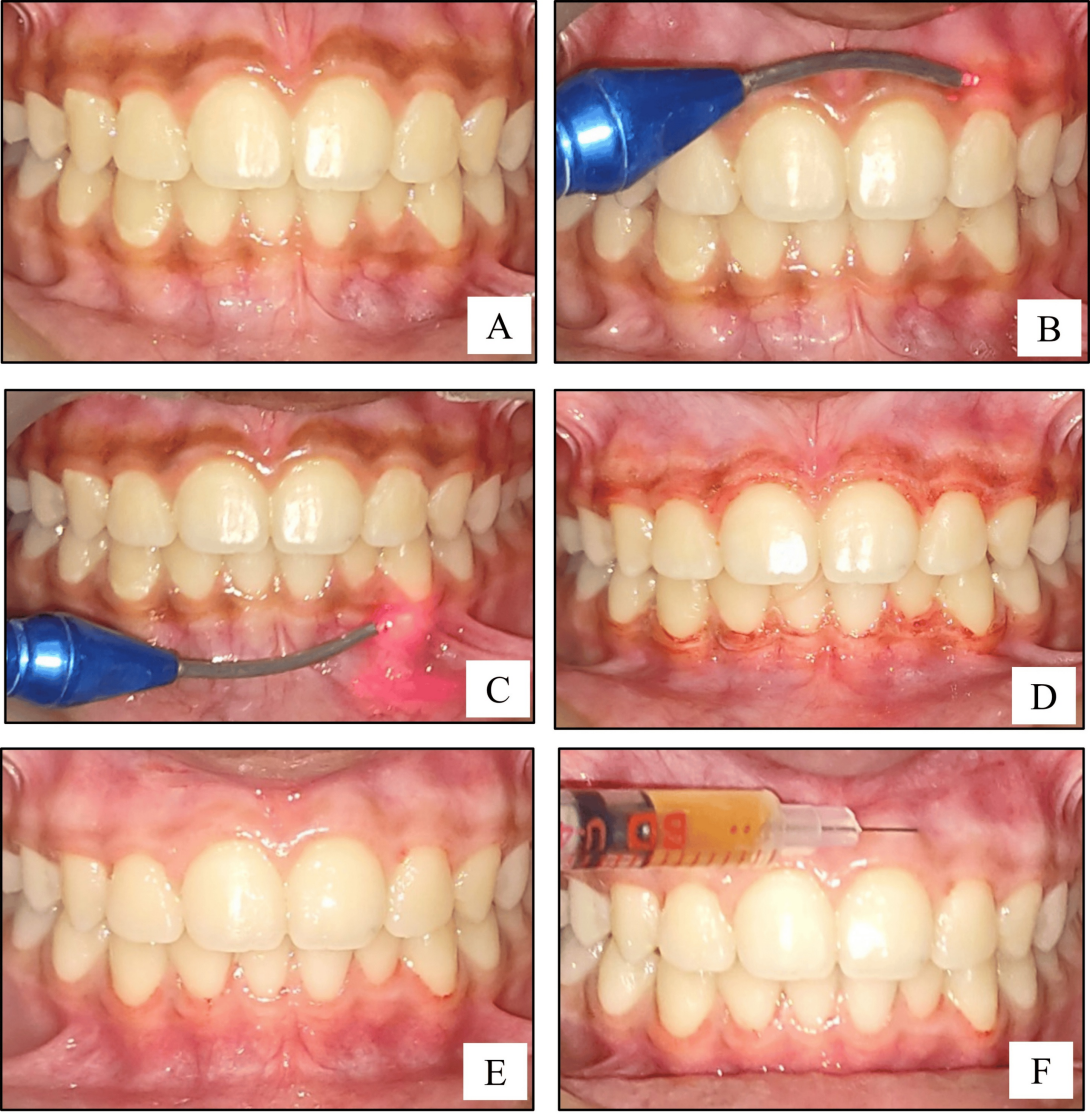
Surgical procedure (laser technique): (A) intraoral baseline photograph showing generalized melanin pigmentation; (B,C) intraoral photograph showing the intraoperative view of depigmentation being performed by the laser technique on maxillary and mandibular gingiva, respectively; (D) intraoral photograph showing immediate postoperative view after depigmentation; (E) intraoral photograph showing two weeks postoperative view after depigmentation; (F) intraoral photograph showing the administration of Vitamin C into the gingiva with the help of an insulin syringe.

**Figure 4:**
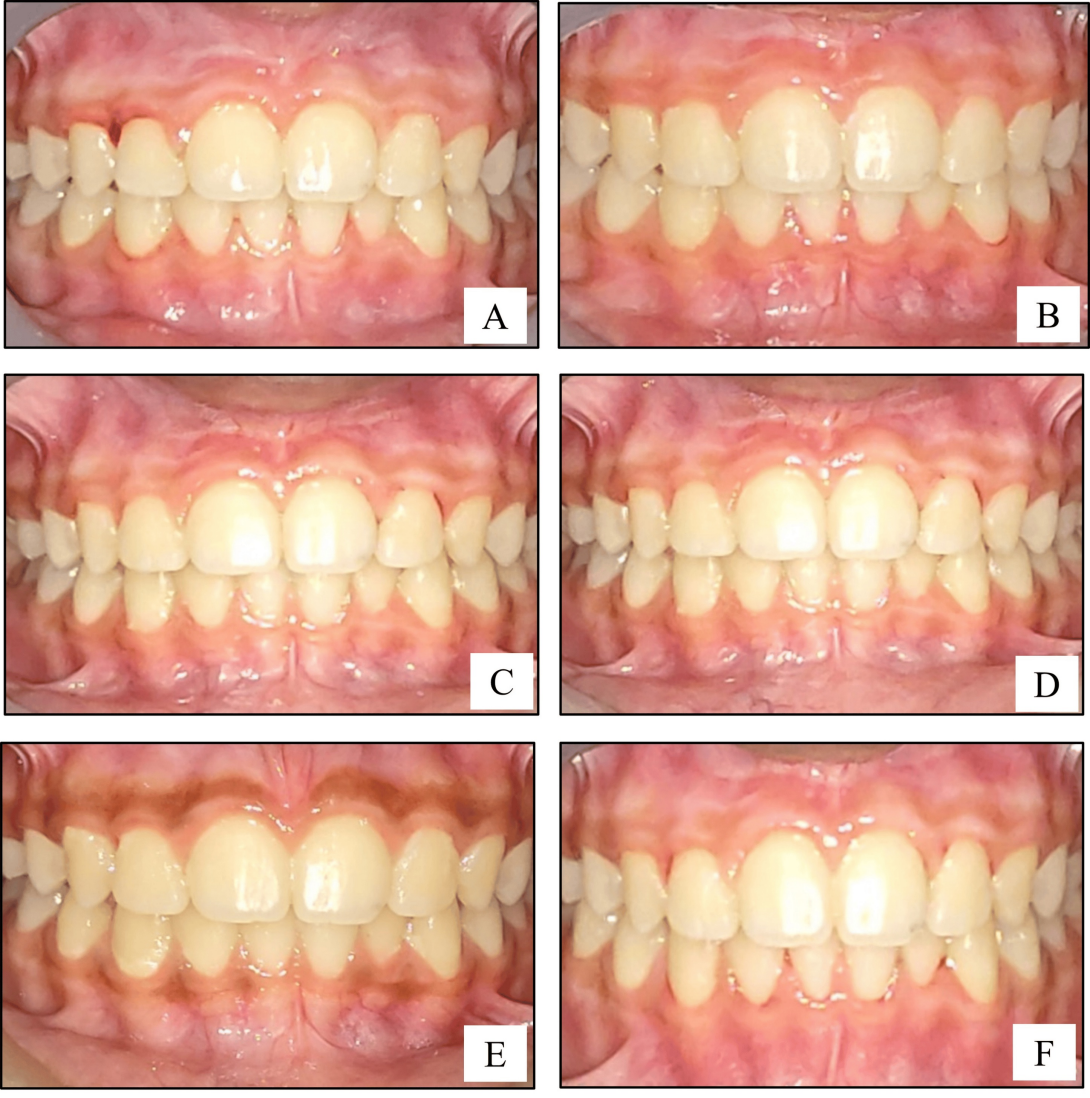
Surgical procedure (laser technique-follow-up): (A) intraoral photograph showing one month postoperative view of the depigmented gingiva; (B) intraoral photograph showing three months postoperative view of the depigmented gingiva; (C) intraoral photograph showing six months postoperative view of the depigmented gingiva; (D) intraoral photograph showing nine months postoperative view of the depigmented gingiva; (E) intraoral photograph showing baseline preoperative view of the pigmented gingiva; (F) intraoral photograph showing one year postoperative view of the depigmented gingiva.

Two weeks after the surgery, the area was anesthetized with local anesthetic spray (Lidocaine USP 15% w/w). Vitamin C was injected into the depigmented sites, Site A2 and Site B2 only (Figures 1F, 2F). About 0.1-0.2 mL of Vitamin C (500 mg/mL) was injected into the gingiva in relation to each tooth, with the help of an insulin syringe keeping in mind the atraumatic procedure. Depending on the area of gingival pigmentation, multiple injections were administered. Subsequently, a similar procedure was repeated after one month and then every month for a period of six months. Photographs were taken during the postoperative follow-up visits of one month, three months, six months, nine months, and one year (Figures 3, 4).

There were no signs of redness (erythema), dryness, or stinging at the sites during and after the administration of Vitamin C. There was no allergic reaction reported to Vitamin C during the administration and also during the follow-up examination. The study commenced on July 21, 2021, and was completed on November 2, 2022.

## Results

All subjects were compliant and reported for the follow-up examination during the study period. The observations of the study were recorded. The data thus obtained was tabulated and subjected to statistical analysis.

Table 1 shows that there was no statistically significant difference seen in the frequencies between the sites (p > 0.05) for age.

**Table 1:** Age distribution of study participants at study sites. There was a statistically non-significant difference seen for the values between the sites (p > 0.05) # indicates non-significance

Sites	Mean ± SD	F-value	p-value of one-way ANOVA
Site A1	26.81 ± 5.95	0.673	0.572^#^
Site A2	24.88 ± 5.40		
Site B1	25.63 ± 5.85		
Site B2	24.38 ± 2.80		

Table 2 shows that there was no statistically significant difference seen in the frequencies between the sites (p > 0.05) in regard to gender.

**Table 2:** Gender distribution of study participants.

Gender	No. of participants
Males	28
Females	36

Table 3 shows that there was no statistically significant difference seen in the frequencies between the sites (maxillary-upper and mandibular-lower) (p > 0.05) in regard to site distribution.

**Table 3:** Site distribution of study participants.

Sites	No. of sites
Maxillary (upper) arch	32
Mandibular (lower) arch	32

Table 4 shows the intersite comparison of the DOPI score. There was a statistically significant difference seen for the DOPI score from one month to one year at the study sites as follows: one month (p = 0.012), three months (p = 0.002), six months (p = 0.000), nine months (p = 0.000), and one year (p = 0.000).

**Table 4:** Intersite comparison of DOPI scores (mean ± SD) assessed at baseline and during the follow-up. DOPI: Dummett-Gupta oral pigmentation index # indicates p-value > 0.05 to be statistically non-significant * indicates p-value < 0.05 to be statistically significant ** indicates p-value < 0.001 to be statistically highly significant

Site assessed	Baseline (B)	2 weeks (2Wks)	1 month (1M)	3 months (3M)	6 months (6M)	9 months (9M)	1 year (1Yr)
Site A1	2.47 ± 0.47	0.00 ± 0.00	0.10 ± 0.15	0.22 ± 0.23	0.52 ± 0.52	0.60 ± 0.55	0.80 ± 0.63
Site A2	2.56 ± 0.51	0.00 ± 0.00	0.00 ± 0.00	0.04 ± 0.15	0.11 ± 0.32	0.21 ± 0.37	0.27 ± 0.42
Site B1	2.31 ± 0.44	0.02 ± 0.06	0.11 ± 0.15	0.27 ± 0.23	0.46 ± 0.49	0.65 ± 0.45	0.71 ± 0.52
Site B2	2.53 ± 0.49	0.00 ± 0.00	0.03 ± 0.12	0.05 ± 0.12	0.10 ± 0.15	0.12 ± 0.15	0.18 ± 0.21
Chi-squared value	2.525	3.000	10.988	15.243	18.032	21.745	19.341
p-value of Kruskal-Wallis test	0.471^#^	0.392^#^	0.012^*^	0.002^**^	0.000^**^	0.000^**^	0.000^**^

Table 5 shows the intersite comparison of the VAS score. There was a statistically highly significant difference seen for the VAS score between sites at Site A1, Site A2, Site B1, and Site B2 at baseline (p = 0.000), one hour (p = 0.000), and two weeks (p = 0.000). There was a statistically significant difference seen for Site A2 and Site B2 at one month (p = 0.000). However, there was a statistically non-significant difference seen for Site A2 and Site B2 at three months (p = 0.317) and six months (p = 1.000).

**Table 5:** Intersite comparison of VAS scores (mean ± SD) for pain assessed at baseline and during the follow-up. VAS: visual analog scale # indicates p-value > 0.05 to be statistically non-significant * indicates p-value < 0.05 to be statistically significant ** indicates p-value < 0.001 to be statistically highly significant

Site assessed	Baseline (B)	1 hour (1Hr)	2 weeks (2Wks)	1 month (1M)	3 months (3M)	6 months (6M)
Site A1	2.56 ± 0.51	4.56 ± 0.51	0.75 ± 0.44	-		
Site A2	2.31 ± 0.47	4.19 ± 0.40	1.56 ± 0.51	1.00 ± 0.00	0.06 ± 0.25	0.00 ± 0.00
Site B1	2.00 ± 0.00	3.06 ± 0.25	0.38 ± 0.50	-		
Site B2	2.00 ± 0.00	3.56 ± 0.51	1.13 ± 0.34	0.38 ± 0.50	0.00 ± 0.00	0.00 ± 0.00
Chi-squared value	20.520	42.275	31.448	14.091	1.000	0.000
p-value of Kruskal-Wallis test	0.000^**^	0.000^**^	0.000^**^	0.000^**^	0.317^#^	1.000^#^

Table 6 shows the intersite comparison of the surface area of pigmentation. There was a statistically significant difference seen for the surface area of pigmentation between sites at two weeks (p = 0.015), one month (p = 0.000), three months (p = 0.000), six months (p = 0.000), nine months (p = 0.001), and one year (p = 0.000) as compared to baseline.

**Table 6:** Intersite comparison of the surface area of pigmentation (mean ± SD) measured in centimeters assessed at baseline and during the follow-up. # indicates p-value > 0.05 to be statistically non-significant * indicates p-value < 0.05 to be statistically significant ** indicates p-value < 0.001 to be statistically highly significant

Site assessed	Baseline (B)	2 weeks (2Wks)	1 month (1M)	3 months (3M)	6 months (6M)	9 months (9M)	1 year (1Yr)
Site A1	4.93 ± 1.79	0.06 ± 0.16	0.12 ± 0.22	0.36 ± 0.38	0.77 ± 0.91	1.01 ± 1.01	1.38 ± 1.16
Site A2	5.63 ± 1.80	0.01 ± 0.03	0.00 ± 0.01	0.07 ± 0.14	0.18 ± 0.32	0.35 ± 0.37	0.43 ± 0.42
Site B1	5.07 ± 1.82	0.09 ± 0.19	0.25 ± 0.31	0.58 ± 0.59	0.96 ± 0.79	1.37 ± 0.96	1.65 ± 1.15
Site B2	5.44 ± 1.83	0.05 ± 0.15	0.07 ± 0.17	0.12 ± 0.21	0.20 ± 0.31	0.29 ± 0.34	0.38 ± 0.40
Chi-squared value	1.422	10.449	23.166	19.750	20.822	17.150	20.229
p-value of Kruskal-Wallis test	0.700^#^	0.015^*^	0.000^**^	0.000^**^	0.000^**^	0.001^**^	0.000^**^

## Discussion

Melanin pigmentation may appear in the gingiva as early as three hours after birth [13]. The attached gingiva is the most frequently pigmented intraoral tissue, followed by the papillary gingiva and the alveolar mucosa [14]. “Gingival melanin hyperpigmentation is caused due to excessive melanin deposition by the melanocytes located in the basal and supra-basal layers of the epithelium” [15]. Although physiological gingival melanin hyperpigmentation does not indicate a medical problem, “black gums” may be a cause of esthetic concern, especially in individuals with a high smile line [5].

No study has been reported on the administration of Vitamin C to prevent repigmentation of gingiva after surgical depigmentation, except for a single case reported by Sheel et al. [16]. This highlighted a great opportunity for research, where understanding the role of Vitamin C could lead to new insights in the field of cosmetic dentistry. Hence, the present study was conducted to evaluate the efficacy of Vitamin C in preventing the repigmentation of gingiva after surgical depigmentation.

Age, gender, and site distribution

It was observed that there was no significant difference in the age, gender, and site distribution of gingival pigmentation. The findings of the present study were similar to those reported by Prasad et al. [14], Ciçek and Ertaş [17], and Yeh [18]. Hyperpigmentation does not exhibit gender predilection and may occur at all ages and races [19]. Melanin pigmentation occurs in the upper and lower arches [20].

DOPI scores

There was a statistically significant decrease in the DOPI scores at the sites treated with scalpel depigmentation along with Vitamin C administration from one month to one year postoperatively at Site A2 as compared to sites treated with scalpel depigmentation alone at Site A1. The findings of the present study are similar to those reported by Shimada et al. [21], Sheel et al. [16], and Mostafa and Alotaibi [22].

The results of the present study were also in accordance with a series of studies conducted by Yussif et al. [7,23,24], wherein they established the role of injectable Vitamin C in the reduction of gingival melanin hyperpigmentation. However, Vitamin C was used as a non-surgical depigmentation agent in contrast to the present study, where Vitamin C was administered postoperatively after surgical scalpel or laser depigmentation so as to prevent the recurrence of pigmentation.

There was a statistically non-significant decrease in DOPI scores between the sites treated with a scalpel or laser depigmentation alone, i.e., Site A1 or Site B1. The findings of the present study are similar to those reported by Kaushik et al. [25], Raaman et al. [26], Suragimath et al. [27], Chhina et al. [28], Inasu and Thomas [29], and Haider et al. [30]. The findings of the present study are similar to those reported by Sanadi and Deshmukh [31] and Kadri and Sanadi [32]. However, Vitamin C was used as a non-surgical depigmentation agent, in contrast to the present study, where Vitamin C was administered after surgical depigmentation.

VAS scores for pain

There was a statistically highly significant decrease seen for the VAS score between the sites treated with laser depigmentation alone, i.e., Site B1, as compared to scalpel depigmentation alone, i.e., Site A1 at baseline and after one hour and two weeks postoperatively. The findings of the present study are similar to those reported by Bhardwaj et al. [33], Mahajan et al. [34], Kaushik et al. [25], Sanadi et al. [35], Raaman et al. [26], Suragimath et al. [27], Chhina et al. [28], and Haider et al. [30].

The findings of the present study are in contrast with those reported by Sharma et al. [36] and Inasu and Thomas [29]. Sharma et al. [36] observed minimal bleeding during surgery, but patient discomfort and pain during the initial healing period were more in electrocautery and laser procedures.

Surface area of pigmentation

There was a statistically significant decrease in the surface area of pigmentation from baseline to one year within the study sites and a statistically significant decrease in the surface area of pigmentation between the sites from baseline to one year postoperatively.

The findings of the present study are similar to those reported by Raaman et al. [26]. They conducted a study to compare the effectiveness of scalpel and laser techniques for depigmentation. They used a VAS to rate the pain and image analysis software ImageJ to compare the rate of gingival repigmentation during a follow-up period of three months. Twenty-five individuals underwent depigmentation with a scalpel, and 25 individuals underwent depigmentation with a laser. They observed lesser VAS scores for pain in individuals treated with the laser technique as compared to the scalpel technique. However, when the rate of repigmentation was assessed, the results were similar with both techniques. The findings of the present study are in contrast to those reported by Suragimath et al. [27] and Perlmutter and Tal [37]. Suragimath et al. [27] observed that the repigmentation was faster in the scalpel technique as compared to the laser technique. The observation of the present study suggests a positive correlation between the administration of Vitamin C along with surgical depigmentation and the prevention of repigmentation after surgical depigmentation.

Limitations of the study

Patient compliance in reporting for follow-up is a major limitation of the study. The duration of the study of one-year follow-up may be insufficient as the evidence for repigmentation varies from four months to 10 years. Hence, long-term follow-up of the patient is essential. Age-related changes and hormonal changes in the occurrence of pigmentation especially in female patients need to be considered.

## Conclusions

Vitamin C is an essential nutrient that plays a vital role in various bodily functions with a significant impact. It has antioxidant properties, neutralizing the free radicals and protecting against cell damage and oxidative stress. It boosts the production of white blood cells and improves the immune function. It helps in collagen production, which is a protein that gives structure to skin, bones, and connective tissue. It enhances the absorption of iron from plant-based foods. It assists in reducing inflammation and improving blood vessel function. It is known to enhance skin tone by reducing melanin production at the molecular level. It helps improve skin hydration by increasing the production of hyaluronic acid.

The findings of the present study can be summarized as laser depigmentation was more beneficial as compared to the scalpel technique in terms of enhanced patient compliance due to minimal pain and discomfort. Secondly, the study also concludes that the administration of Vitamin C is an effective, safe, simple, and minimally invasive technique to reduce the reoccurrence of melanin pigmentation of the gingiva after surgical depigmentation. This may eliminate the need for a repeat surgical procedure.

## References

[1] Elavarasu S, Thangavelu A, Alex S (2015). Comparative evaluation of depigmentation techniques in split-mouth design with electrocautery and laser. J Pharm Bioallied Sci.

[2] Itoiz ME, Carranza FA (2002). The gingiva. Carranza’s Clinical Periodontology.

[3] Trelles MA, Verkruysse W, Segui JM, Udaeta A (1993). Treatment of melanotic spots in the gingiva by argon laser. J Oral Maxillofac Surg.

[4] Dummett CO (1985). Pertinent considerations in oral pigmentations. Br Dent J.

[5] Lin YH, Tu YK, Lu CT, Chung WC, Huang CF, Huang MS, Lu HK (2014). Systematic review of treatment modalities for gingival depigmentation: a random-effects Poisson regression analysis. J Esthet Restor Dent.

[6] Roshna T, Nandakumar K (2005). Anterior esthetic gingival depigmentation and crown lengthening: report of a case. J Contemp Dent Pract.

[7] Yussif NM, Zayed SO, Hasan SA, Sadek SS (2016). Evaluation of injectable vitamin C as a depigmenting agent in physiologic gingival melanin hyperpigmentation: a clinical trial. Rep Opinion.

[8] Padayatty SJ, Levine M (2016). Vitamin C: the known and the unknown and Goldilocks. Oral Dis.

[9] Telang PS (2013). Vitamin C in dermatology. Indian Dermatol Online J.

[10] Dummett CO, Gupta OP (1964). Estimating the epidemiology of oral pigmentation. J Natl Med Assoc.

[11] Matthews DC, McCulloch CA (1993). Evaluating patient perceptions as short-term outcomes of periodontal treatment: a comparison of surgical and non-surgical therapy. J Periodontol.

[12] Schneider CA, Rasband WS, Eliceiri KW (2012). NIH Image to ImageJ: 25 years of image analysis. Nat Methods.

[13] Dummett CO (1946). Physiologic pigmentation of the oral and cutaneous tissues in the Negro. J Dent Res.

[14] Prasad SSV, Agrawal N, Reddy NR (2010). Gingival depigmentation: a case report. People’s J Sci Res.

[15] Dummett CO, Barens G (1971). Oromucosal pigmentation: an updated literary review. J Periodontol.

[16] Sheel V, Purwar P, Dixit J, Rai P (2015). Ancillary role of vitamin C in pink aesthetics. BMJ Case Rep.

[17] Ciçek Y, Ertaş U (2003). The normal and pathological pigmentation of oral mucous membrane: a review. J Contemp Dent Pract.

[18] Yeh CJ (1998). Cryosurgical treatment of melanin pigmented gingiva. Oral Surg Oral Med Oral Pathol Oral Radiol Endod.

[19] Yoo JM, Park HJ, Choi SW, Kim HO (2001). Vitamin C-iontophoresis in melasma. Korean J Dermatol.

[20] Verma M, Khan MA, Pathak AK, Lal N, Kaushal S, Haque AU (2022). Gingival depigmentation using simple de-epithelization by bur abrasion technique-a case report. Int J Adv Res.

[21] Shimada Y, Tai H, Tanaka A, Ikezawa-Suzuki I, Takagi K, Yoshida Y, Yoshie H (2009). Effects of ascorbic acid on gingival melanin pigmentation in vitro and in vivo. J Periodontol.

[22] Mostafa D, Alotaibi SM (2022). A successful esthetic approach of gingival depigmentation using microneedling technique and ascorbic acid (vitamin C). Case Rep Dent.

[23] Yussif NM, Korany NS, Abbass MS (2017). Evidence of the effect of intraepidermic vitamin C injection on melanocytes and keratinocytes in gingival tissues: in vivo study. Dentistry.

[24] Yussif NM, Abdel Rahman AR, Elbarbary A (2019). Minimally invasive non-surgical locally injected vitamin C versus the conventional surgical depigmentation in treatment of gingival hyperpigmentation of the anterior esthetic zone: a prospective comparative study. Clin Nutr Exp.

[25] Kaushik N, Srivastava N, Kaushik M, Gaurav V (2013). Efficacy of different techniques of gingival depigmentation: a comparative evaluation with a case report. Int J Laser Dent.

[26] Raaman AR, Pratebha B, Jananni M, Saravanakumar R (2016). Comparison of efficacy of depigmentation of gingiva in terms of ImageJ intensity values and surface area of repigmentation using scalpel and diode laser. Int J Oral Health Sci.

[27] Suragimath G, Lohana MH, Varma S (2016). A split mouth randomized clinical comparative study to evaluate the efficacy of gingival depigmentation procedure using conventional scalpel technique or diode laser. J Lasers Med Sci.

[28] Chhina S, Gakhar A, Gupta S, Sharma SE, Arora SA (2019). Assessment of clinical outcomes and patient response to gingival depigmentation by scalpel surgical stripping and diode laser: a randomized split-mouth study. J Adv Oral Res.

[29] Inasu S, Thomas B (2021). A pink smile: depigmentation using diode laser and surgical scalpel. Int J Experiment Dent Sci.

[30] Haider K, Hussain S, Kumar V, Shakti P (2022). Comparative evaluation of gingival depigmentation by scalpel, electrocautery and diode laser. Neuroquantology.

[31] Sanadi RM, Deshmukh RS (2021). Vitamin c as a non-surgical gingival depigmentation agent-case study. Med Res Chronicles.

[32] Kadri KA, Sanadi RM (2021). Evaluation of injectable vitamin C as a depigmenting agent in physiological gingival melanin hyperpigmentation: an analytical study. Int J Recent Sci Res.

[33] Bhardwaj A, Grover HS, Lal S (2012). Gingival depigmentation with scalpel and diode laser. World J Dent.

[34] Mahajan V, Dodwad V, Nagpal S (2013). Gingival depigmentation: a case series. J Dent Spec.

[35] Sanadi RM, Suthar N, Bhusari BM, Chelani L (2015). Gingival depigmentation using scalpel technique versus laser technique: a case report. J Dent Med Sci.

[36] Sharma T, Agrawal C, Shah M (2020). An overview on gingival depigmentation procedures: case series and follow up. Acta Scientific Dent Sci.

[37] Perlmutter S, Tal H (1986). Repigmentation of the gingiva following surgical injury. J Periodontol.

